# Regaining Versus Not Regaining Function Following Hip Fracture—A Descriptive Study

**DOI:** 10.3390/geriatrics4010021

**Published:** 2019-02-14

**Authors:** Caspar Hansen, Dorte Melgaard

**Affiliations:** 1 Physio- and Occupational Therapy Department, North Denmark Regional Hospital, DK-9800 Hjørring, Denmark; dmk@rn.dk; 2 Center for Clinical Research, North Denmark Regional Hospital, DK-9800 Hjørring, Denmark

**Keywords:** rehabilitation, elderly, basic mobility, cumulated ambulation score, hip fracture, physiotherapy

## Abstract

The aim of this study was to study the prevalence of patients who did not regain pre-fracture basic mobility status (PF-BMS) at a task-specific level at discharge with 6-month follow-up. Furthermore, the objective was to make a comparative description between patients who did and did not regain PF-BMS measured with the Cumulated Ambulation Score (CAS). A cross-sectional study with follow-up at discharge and 6 months was performed from June 2015 to November 2017. Inclusion criteria: all patients ≥65 years admitted with first-time hip fracture. In all, 235 patients were included in the analyses at discharge (76% female, median age 85 (83–87)) and 59 patients at 6 months (48% female, median age 82 (75–88)). At discharge, getting in/out of bed had the highest prevalence of non-regained ability. At 6 months this was the case for getting in/out of bed and walking. At discharge, significant between-group differences were found regarding age, pre-fracture function (PFF), dementia, pre-fracture residence (PFR), comorbidity, and length of stay (LOS). At follow-up, significant differences in PFF, PFR, discharge destination (DD) and residence at 3 months after discharge (RES-3) were found. Getting in/out of bed was the most difficult task to regain both during admission and long term.

## 1. Introduction

Hip fracture is a common occurrence worldwide in the geriatric population, with incidences of 150–250/100,000 in developed countries [1]; it is considered to cause a substantial socioeconomic burden [2,3,4]. Furthermore, sustaining a hip fracture has great consequences for the individual in terms of increased long-term mortality [5,6] and disability in terms of reduced physical performance in terms of general mobility in their own home and in the community [6,7]. Loss of function directly related to the hip fracture has been estimated to be 15–20% at 12 months after injury [8]. Functional decline occurs in different levels of the patients’ everyday lives and influences both advanced activities of daily living (ADL) as well as basic ADLs such as getting in and out of bed, rising from a chair, and walking [8]. Not regaining pre-fracture basic mobility status (PF-BMS) upon hospital discharge has proven to be an independent risk factor for 1- and 5-year mortality [9]. It has been reported that up to 77% of patients regain their PF-BMS at discharge [10] and that the bulk of functional recovery occurred during the first 6 months after discharge for patients who had not fully recovered their function at discharge [11,12]. However, it remains undescribed which basic mobility tasks have the highest prevalence of patients not regaining PF-BMS at discharge as well as after post-discharge rehabilitation. Additionally, comparative descriptions of patients who do not regain PF-BMS and those who do are sparse.

A measurement of basic mobility status (BMS) can be obtained by using the Cumulated Ambulation Score (CAS), which is easy to apply, reliable, and standardized [13,14]. The CAS is a composite score describing the patient’s independence in three tasks of basic mobility following hip fracture: (1) getting in and out of bed, (2) rising from a chair, and (3) walking with an appropriate walking aid [13]. Each task is scored from 0–2, resulting in a total score of 0–6 [13]. The score 0 is given if the patient is unable to perform the task even with human assistance, 1 if the patient is able to perform the task with physical or verbal support from another person, and 2 if the patient is able to perform the task independently with or without the use of assistive aids [13]. A total score of 6 indicates total independence in basic mobility [13]. 

To aid clinicians in applying rehabilitative interventions aimed at the tasks most difficult for the patients a study of the prevalence of not regaining PF-BMS in the tasks of CAS is warranted. 

The primary aim of this observational study was to study the prevalence of patients who did not regain PF-BMS at a task-specific level at hospital discharge and following post-discharge rehabilitation. The secondary aim was to make a comparative description of patients who did not regain task specific PF-BMS and those who did.

## 2. Materials and Methods 

**Figure 1:**
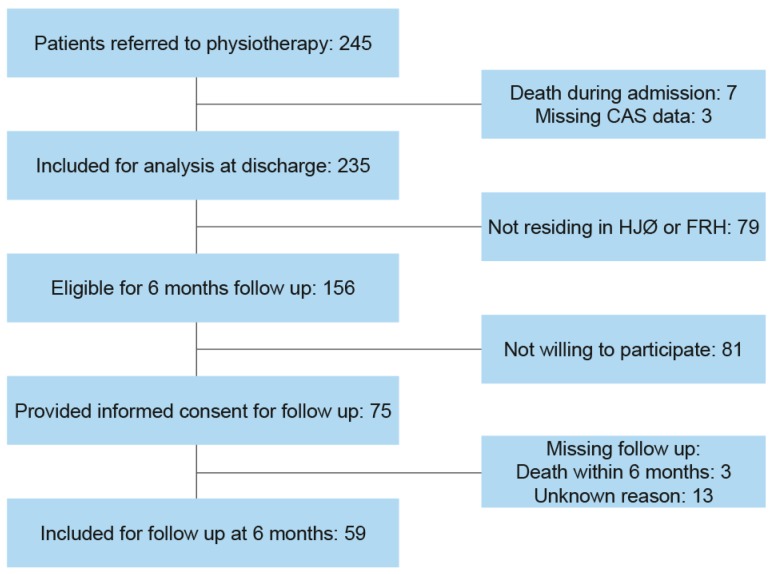
Flowchart of included and excluded patients. HØJ = Hjøring municipality, FRH = Frederikshavn municipality, CAS = Cumulated Ambulation Score.

A cross-sectional observational study with longitudinal follow up was conducted in the period from June 2015 to November 2017. Follow up was conducted at discharge and 6 months after discharge. We included all patients ≥65 years with first-time hip fracture admitted to the Department of Orthopedic Surgery at North Denmark Regional Hospital. A total of 245 consecutive patients were included and referred to physiotherapy during admission. Of those, 235 (96%) patients were included for analysis at discharge and 59 (24%) patients participated in the follow up at 6 months after discharge. Reasons for exclusion are illustrated in Figure 1. 

Patients’ age, gender, pre-fracture residence (PFR), and fracture type were obtained via patients’ charts during hospital admission. Fracture types were registered as medial, pertrochanteric, and subtrochanteric fracture. The presence of comorbidity based on hospital diagnosis within 5 years of admission was evaluated using the Charlson Comorbidity Index (CCI) [15]. Length of stay (LOS) and time to surgery (TTS) were defined as time from admission to time of discharge from the orthopedic ward and time of onset of surgery. LOS and TTS were calculated based on registered times in patients’ charts. PF-BMS was self-reported through interviews by experienced physiotherapists in the orthopedic ward. If the patient was not able to provide valid information in the interview, data regarding PF-BMS were obtained from hospital records or from the patient’s relatives. Information about whether or not the patient had been diagnosed with dementia was obtained from hospital records. Additional descriptive data were obtained for follow up at 6 months after discharge and were: discharge destination (DD), place of residence at 3 months (RES-3), place of rehabilitation, and rehabilitation setting. DD and RES-3 was recorded as own home versus institutional care. Options for place of rehabilitation were own home or rehabilitation center. The possible rehabilitation settings were individual training or group training. The additional descriptive data were attained from municipal rehabilitation charts and supplied by physiotherapists from the Hjørring (HJØ) and Frederikshavn (FRH) municipalities.

The outcome of interest was “not regaining” of PF-BMS in the three tasks of the CAS: (1) getting in and out of bed, (2) rising from a chair, and (3) walking with an appropriate walking aid. CAS was assessed through observation and recorded by experienced physiotherapists in the orthopedic ward on the day of discharge as part of daily practice. The outcome was defined as “not regained” if CAS at follow up was less than PF-BMS. Information regarding CAS at 6 months (CAS-6) was attained through observation and recorded by experienced physiotherapists from Hjørring or Frederikshavn municipality during a home visit as close to six months after discharge as possible, to observe the patients perform the tasks of CAS and record the score for each task and the total score.

### 2.1. Procedure

Mobilization was initiated on the day of surgery if possible, ideally within 24 h after surgery. Full weight bearing was allowed unless the surgeon prescribed other specific regimes. On the first postoperative day, intensive physiotherapy was initiated, comprising daily strengthening exercises as well as functional exercises and mobilization on weekdays. Nursing staff continued mobilization on weekends. At discharge, patients were issued a rehabilitation plan to ensure further municipal rehabilitation. The default time frame was three months but could finish before three months if the patient’s goal had been reached or extended if necessary. The rehabilitation program consisted of two individual or group training sessions per week with a physiotherapist and took place in the patient’s own home or as an out-patient program in a rehabilitation center. Some patients started individual rehabilitation but were later transferred to group rehabilitation thus receiving rehabilitation in both settings. The rehabilitation program was initiated within five days after discharge at the latest. 

### 2.2. Statistical Analysis

Descriptive statistics of the demographic variables include the number and percentage of patients for categorical variables, and median (IQR) for continuous variables. When comparing two groups, differences between groups were analyzed using Fisher’s exact test for categorical variables and the Wilcoxon rank-sum test for continuous data.

TTS was dichotomized to surgery <24 h versus ≥24 h after admission. This was in accordance with previous studies documenting the influence of timing of surgery on patients’ functional abilities following hip fracture [16,17]. Comorbidity measured with CCI was dichotomized to presence of comorbidity (CCI > 0) versus no comorbidity (CCI = 0). PF-BMS was dichotomized to independent (CAS = 6) versus dependent (CAS < 6).

Data has been analyzed as descriptive comparison of groups of patients who have regained or not regained basic mobility, and follow up on group basis.

The significance level was <0.05. The STATA 14.0 (StataCorp, College Station, TX, USA) software package was used for data analysis.

### 2.3. Ethics and Registration

The study was reported to the Danish Data Protection Agency under the coverage of the general notification from the North Denmark Region-Scientific Health Research in The North Denmark Region (2008-58-0028). The study’s identification number is 2015-88. Collection, management and handling of data related to 6-months follow up were conducted in accordance with the Helsinki declaration [18]. 

## 3. Results

A total of 235 patients were included in the analyses at discharge (76% female, median age 85 (83–87)) and 59 patients were included in the analyses at 6 months after discharge (48% female, median age 82 (75–88)). Baseline characteristics of all patients are shown in Table 1. Medial fractures had occurred in 121 patients (51.5%), pertrochanteric fractures in 94 patients (40%), and subtrochanteric fractures in 20 patients (8.5%). There were no differences between per- and subtrochanteric fractures regarding the outcome variables, and they were pooled for analyses. 

At discharge, getting in and out of bed was the task with the highest prevalence of not regaining PF-BMS with 55% (*n* = 129) not regaining their PF-BMS. In total 52% (*n* = 123) did not regain their PF-BMS in walking and 44% (*n* = 103) did not regain their PF-BMS in rising from a chair. At 6 months after discharge, getting out of bed as well as walking had the highest prevalence of not regaining PF-BMS with 10% (*n* = 6). In total, 8% (*n* = 5) did not regain their PF-BMS in rising from a chair.

At discharge, there were significant difference between the two groups in the task of getting in and out of bed regarding age, pre-fracture function, presence of dementia and their PFR as illustrated in Table 2. For the tasks of “rising from a chair” and “walking”, significant differences were found regarding age, presence of comorbidity, length of stay, presence of dementia, and PFR. 

The differences between groups at 6-months follow up are illustrated in Table 3. None of the observed differences between the groups that proved to be significant at discharge remained significant 6 months after discharge for the task of getting in and out of bed. The only significant difference for this task was RES-3. Same pattern was found for the task of rising from a chair. None of the significant differences at discharge remained significant at 6 months. In addition to RES-3, a significant difference in discharge destination was observed between the two groups. For the task of walking the significant difference in PFR remained significant at 6 months. Furthermore, a significant difference in pre-fracture function as well as in RES-3 was detected.

## 4. Discussion

The primary aim of the current study was to examine the prevalence of not regaining PF-BMS at discharge and at 6 months after discharge. The CAS activity of getting in and out of bed had the highest prevalence of not regaining PF-BMS, followed by walking and rising from a chair. At 6 months after discharge, getting in and out of bed still had the highest prevalence of not regaining PF-BMS. The secondary aim was to make a comparative description between patients who do not regain task specific PF-BMS and those who do.

We found the CAS activity of getting in and out of bed to have the highest prevalence of not regaining PF-BMS followed by walking and rising from a chair. The same ranking was found in a recent study [9]. However, the prevalence of not regaining PF-BMS for each of the CAS activities found by Kristensen et al. [9] was higher than in the present study. This discrepancy could be explained by the exclusion of non-independent patients in the study by Kristensen et al. [9], whereas non-independent patients are included in the present study.

At discharge there were significant differences between the two groups regarding age, presence of dementia and PFR for all three tasks of CAS. Older age has been associated with not regaining PF-BMS at discharge in previous studies [10,19], and thus supports the descriptive differences regarding age found in the present study. The difference in the presence of dementia in the two groups is supported by a study by Buecking et al. which showed that an increasing Mini-Mental State Examination score was associated with improved functional outcome [20]. Another study by Kristensen et al. shows that low mental status is crudely associated with not regaining independent BMS [21]. Information regarding dementia was obtained from hospital records. Thus, it is possible that the prevalence of 18.3% for dementia in the present study is underreported. However, results from a meta-analysis comprising five studies from Sweden, Spain and Italy and a total of 1,500 patients with a prevalence of 19.2% [22] supports the prevalence from the present study. 

There was a significant difference in PFR in each of the CAS activities. Of the 235 patients included for analyses at discharge, 79 (33.6%) were admitted from institutional care. Similar proportions of 42.7% [7] and 27% [23] have been reported in other studies. In support of the observed difference in the present study, Kammerlander et al. has previously shown that patients residing in a nursing home before sustaining a hip fracture have a significant lower Barthel Index and Parker Score than patients admitted from their own homes [7]. 

Additionally there were significant differences in regaining PF-BMS in the tasks of rising from a chair as well as walking regarding the presence of comorbidity and LOS. The degree of comorbidity has previously been established as a predictor of reducing the ability to stand and to walk within the first 2–4 days post-surgery [20]. However, in the present study, data regarding CCI did not resemble a normal distribution and was therefore treated as a binary variable. Hence not indicating the degree of comorbidity but merely whether or not comorbidity was present. CCI was recorded from hospital charts and CCI is calculated based on hospital diagnoses within the last 5 years prior to admission. These factors impose a risk of CCI being underreported. 

In the current study a significant difference in LOS was observed regarding the tasks of rising from a chair and walking. Guccione et al. found that increased LOS improved function in tasks similar to the ones used in the present study and this supports our findings of shorter LOS for patients not regaining PF-BMS [24]. The shorter LOS for patients not regaining PF-BMS can explained by the difference in comorbidity between the two groups. An acute need for postoperative treatment of comorbidities may have led to a transfer to the hospital’s medical ward. This type of case would have been registered as a discharge from the orthopedic ward before actual hospital discharge. Data regarding transfers to medical wards have not been obtained for this study. Another explanation could be the difference in PFR. Significantly shorter LOS for patients from long-term care (LTC) has previously been described and the same study reported a significantly smaller proportion of patients from LTC regaining their pre-fracture function [25]. 

Six patients had not regained PF-BMS at 6-months follow up. Due to the small group size, Fisher’s exact test was used when comparing the two groups.

At 6-months follow up, RES-3 was the only variable with significant difference between the two groups in all three tasks of CAS. Besides the present study, no studies comparing post discharge residence to long-term function have been identified. Hence, a direct comparison of the finding in relation to existing literature is not possible. However, Beupre et al. identified a difference in regaining pre-fracture function between community dwelling patients and patients from LTC at 6-months following hip fracture, especially regarding walking and transfer activities [25].

A significant difference in pre-fracture function regarding regaining walking ability was found in the present study. To support this result, a study by Pioli et al. found that high pre-fracture ADL-abilities were significantly associated with regaining pre-fracture walking ability 6-months following hip fracture [26]. Additionally, Vochteloo et al. found a significantly higher proportion of hip fracture patients with low pre-fracture ADL amongst those who did not regain their walking ability compared to patients with high pre-fracture ADL [27]. 

One of the strengths of the current study is that all patients admitted during the inclusion period were consecutively enrolled and included in the National Database of Hip Fractures. Hence, it is considered unlikely that any selection bias of enrolled patients has occurred.

Another strength of this study is that data regarding outcome are obtained by experienced physiotherapists using a reliable and validated measure. The scoring of CAS follows standardized guidelines, does not require calibration and, as such, does not pose a threat to internal validity. The physiotherapists assessing CAS at discharge were also training the patients on a daily basis and hence not blinded. However, the threat of bias due to lack of blinding is reduced by the specific guidelines for assigning scores in CAS.

A weakness of the present study is the relatively large loss to follow up, constituting 62% of the eligible patients. A possible explanation for this loss can be found in the time and the context in which the informed consent was obtained. It can be difficult for elderly patients to consider whether or not to participate in follow up in 6 months shortly after the trauma of sustaining a hip fracture and going through surgery. 

### Implications for Practice

At discharge, getting in and out of bed was identified as the activity with the highest prevalence of not regaining PF-BMS, followed by walking and rising from a chair. This aids clinicians working in the acute rehabilitation in choosing which interventions to initiate in the early phase of rehabilitation following hip fracture. Getting in and out of bed was still the task with highest prevalence of not regaining PF-BMS at 6 months. Previous studies have highlighted the importance of getting out of bed following hip fracture surgery and early mobilization is associated with reduced mortality and risk of readmission [28], as well as increased independence following hip fracture surgery [29]. 

All variables in which a significant difference was observed were non-modifiable factors. Hence, it is not possible to affect these factors through postoperative rehabilitation. However, the new and useful information which can be derived from this study is that patients presenting with advanced age, non-independent PFF, dementia, admission from institutional care, or comorbidities are at risk of not regaining PF-BMS and should receive extra attention. In the post-discharge rehabilitation extra attention should be given to those who are discharged to institutional care and those in institutional care 3 months after discharge. 

Studies investigating associations between regaining PF-BMS at a task-specific level and postoperative pain, nosocomial infections, extent of rehabilitation, and other modifiable factors are warranted. Furthermore, studies investigating the association between regaining PF-BMS and mortality as well as readmission would be of interest.

## Figures and Tables

**Table 1:** Baseline demographics and clinical characteristics.

	At Discharge (*n* = 235)	At 6 Months (*n* = 59)
Gender		
Female	178 (76%)	48 (81%)
Male	57 (24%)	11 (19%)
Age-years	85 (83–87)	82 (75–88)
Fracture type		
Medial	121 (51%)	33 (56%)
Pertrochanteric and subtrochanteric	114 (99%)	26 (44%)
Comorbidity		
Yes	178 (76%)	49 (83%)
No	57 (24%)	10 (17%)
Length of stay-days (LOS)	7.0 (5.0–8.7)	7.8 (6.1–9.3)
Time to surgery		
<24 h	131 (56%)	31 (53%)
>24 h	104 (44%)	28 (47%)
Pre-fracture function (PFF)		
Independent	213 (91%)	56 (95%)
Not independent	22 (9%)	3 (5%)
Dementia		
Yes	43(18%)	6(10%)
No	192 (82%)	53 (90%)
Pre-fracture residence (PFR)		
Own home	156 (66%)	43 (74%)
Institutional care	79 (34%)	15 (26%)
Discharge destination (DD)		
Own home	-	33 (56%)
Institutional care		26 (44%)
Residence at 3 months (RES-3)		
Own home	-	44 (75%)
Institutional care		15 (25%)
Place of rehabilitation *		
Own home	-	21 (36%)
Rehabilitation facility		37 (64%)
Rehabilitation setting *		
Individual	-	46 (79%)
Group		12 (21%)

* Missing data from one patient.

**Table 2:** Comparison of regained pre-fracture basic mobility status vs. not regained pre-fracture basic mobility status at discharge.

	Regained	Not Regained	*p*-Value
Getting in/Out of Bed			
Gender			
Female	78 (44%)	100 (56%)	
Male	28 (49%)	29 (51%)	0.542
Age-years	82 (75–88)	87 (81–91)	<0.001
Fracture type			
Medial	61 (50%)	60 (50%)	
Pertrochanteric and subtrochanteric	45 (39%)	69 (61%)	0.115
Comorbidity			
Yes	75 (42%)	103 (58%)	
No	31 (54%)	26 (46%)	0.127
Length of stay, days (LOS)	7.1 (5.6–9.0)	7.0 (4.8–8.3)	0.094
Time to surgery			
<24 h	61 (47%)	70 (53%)	
>24 h	45 (43%)	59 (57%)	0.692
Pre-fracture function (PFF)			
Independent	88 (41%)	125 (59%)	
Not independent	18 (82%)	4 (18%)	<0.001
Dementia			
Yes	6 (14%)	37 (86%)	
No	100 (52%)	92 (48%)	<0.001
Pre-fracture residence (PFR)			
Own home	86 (55%)	70 (44%)	
Institutional care	20 (25%)	59 (75%)	<0.001
**Rise from a chair**			
Gender			
Female	100 (56%)	78 (44%)	
Male	32 (56%)	25 (44%)	0.558
Age, years	83 (75–89)	87 (81–91)	0.003
Fracture type			
Medial	72 (60%)	49 (40%)	
Pertrochanteric and subtrochanteric	60 (53%)	54 (47%)	0.296
Comorbidity			
Yes	86 (48%)	92 (52%)	
No	46 (81%)	11 (19%)	<0.001
Length of stay-days (LOS)	7.5 (5.9–9.0)	6.1 (4.0–8.0)	<0.001
Time to surgery			
<24 h	74 (56%)	57 (44%)	
>24 h	58 (56%)	46 (44%)	1.000
Pre-fracture function (PFF)			
Independent	117 (55%)	96 (45%)	
Not independent	15 (68%)	7 (32%)	0.266
Dementia			
Yes	7 (16%)	36 (84%)	
No	125 (65%)	67 (35%)	<0.001
Pre-fracture residence (PFR)			
Own home	106 (68%)	50 (32%)	
Institutional care	26 (33%)	53 (67%)	<0.001
**Walking**			
Gender			
Female	83 (47%)	95 (53%)	
Male	29 (51%)	28 (49%)	0.648
Age-years	82 (75–88)	88 (82–92)	<0.001
Fracture type			
Medial	63 (52%)	58 (48%)	
Pertrochanteric and subtrochanteric	49 (43%)	65 (57%)	0.192
Comorbidity			
Yes	73 (41%)	105 (59%)	
No	39 (68%)	18 (32%)	<0.001
Length of stay, days (LOS)	7.2 (5.8–9.1)	6.7 (4.2–8.1)	0.006
Time to surgery			
<24 h	65 (50%)	66 (50%)	
>24 h	47 (45%)	57 (55%)	0.514
Pre-fracture function (PFF)			
Independent	103 (48%)	110 (52%)	
Not independent	9 (41%)	13 (59%)	0.655
Dementia			
Yes	5 (12%)	38 (88%)	
No	107 (56%)	85 (44%)	<0.001
Pre-fracture residence (PFR)			
Own home	94 (60%)	62 (40%)	
Institutional care	18 (23%)	61 (77%)	<0.001

**Table 3:** Comparison of regained pre-fracture basic mobility status vs. not regained pre-fracture basic mobility status at 6-months following discharge.

	Regained	Not Regained	*p*-Value
Getting in/Out of Bed			
Gender			
Female	44 (92%)	4 (8%)	
Male	9 (82%)	2 (8%)	0.310
Age-years	81 (75–88)	85 (70–92)	0.720
Fracture type			
Medial	30 (91%)	3 (9%)	
Pertrochanteric and subtrochanteric	23 (88%)	3 (12%)	1.000
Comorbidity			
Yes	43 (88%)	6 (12%)	
No	10 (100%)	0 (0%)	0.577
Length of stay-days (LOS)	7.8 (6.1–9.2)	7.6 (3.3–11.2)	0.880
Time to surgery			
<24 h	30 (97%)	1 (3%)	
>24 h	23 (82%)	5 (18%)	0.092
Pre-fracture function (PFF)			
Independent	51 (91%)	5 (9%)	
Not independent	2 (67%)	1 (33%)	0.279
Dementia			
Yes	5 (83%)	1 (17%)	
No	48 (91%)	5 (9%)	0.490
Pre-fracture residence (PFR)			
Own home	41 (93%)	3 (7%)	
Institutional care	12 (80%)	3 (20%)	0.172
Discharge destination (DD)			
Own home	31 (94%)	2 (6%)	
Institutional care	22 (85%)	4 (15%)	0.390
Residence at 3 months (RES-3)			
Own home	42 (95%)	2 (5%)	
Institutional care	11 (73%)	4 (27%)	0.032
Place of rehabilitation *			
Own home	17 (81%)	4 (19%)	
Rehabilitation facility	36 (97%)	1 (3%)	0.053
Rehabilitation setting *			
Individual	42 (91%)	4 (9%)	
Group	11 (92%)	1 (8%)	1.000
**Rise from a chair**			
Gender			
Female	45 (94%)	3 (6%)	
Male	9 (82%)	2 (18%)	0.230
Age-years	82 (75–88)	83 (75–88)	0.913
Fracture type			
Medial	31 (94%)	2 (6%)	
Pertrochanteric and subtrochanteric	23 (88%)	3 (12%)	0.646
Comorbidity			
Yes	44 (90%)	5 (10%)	
No	10 (100%)	0 (0%)	0.577
Length of stay-days (LOS)	7.8 (6.1–9.3)	7.0 (3.2–9.2)	0.355
Time to surgery			
<24 h	30 (97%)	1 (3%)	
>24 h	24 (86%)	4 (14%)	0.180
Pre-fracture function (PFF)			
Independent	52 (93%)	4 (7%)	
Not independent	2 (67%)	1 (33%)	0.237
Dementia			
Yes	5 (83%)	1 (17%)	
No	49 (92%)	4 (8%)	0.427
Pre-fracture residence (PFR)			
Own home	41 (93%)	3 (7%)	
Institutional care	13 (87%)	2 (13%)	0.596
Discharge destination (DD)			
Own home	33 (100%)	0 (0%)	
Institutional care	21 (81%)	5 (19%)	0.013
Residence at 3 months (RES-3)			
Own home	43 (98%)	1 (2%)	
Not own home	11 (73%)	4 (27%)	0.013
Place of rehabilitation *			
Own home	19 (90%)	2 (10%)	
Rehabilitation facility	35 (95%)	2 (5%)	0.615
Rehabilitation setting *			
Individual	42 (91%)	4 (9%)	
Group	12 (100%)	0 (0%)	0.571
**Walking**			
Gender			
Female	45 (94%)	3 (6%)	
Male	8 (73%)	3 (27%)	0.072
Age-years	81 (75–88)	85 (70–88)	0.930
Fracture type			
Medial	30 (91%)	3 (9%)	
Pertrochanteric and subtrochanteric	23 (88%)	3 (12%)	1.000
Comorbidity			
Yes	43 (88%)	6 (12%)	
No	10 (100%)	0 (0%)	0.577
Length of stay-days (LOS)	7.8 (6.1–9.2)	7.6 (5.4–10.9)	0.960
Time to surgery			
<24 h	30 (97%)	1 (3%)	
>24 h	23 (82%)	5 (18%)	0.092
Pre-fracture function (PFF)			
Independent	52 (93%)	4 (7%)	
Not independent	1 (33%)	2 (67%)	0.025
Dementia			
Yes	5 (83%)	1 (17%)	
No	49 (92%)	4 (8%)	0.490
Pre-fracture residence (PFR)			
Own home	42 (95%)	2 (5%)	
Institutional care	11 (73%)	4 (27%)	0.034
Discharge destination (DD)			
Own home	32 (97%)	1 (3%)	
Institutional care	21 (81%)	5 (19%)	0.078
Residence at 3 months (RES-3)			
Own home	43 (98%)	1 (2%)	
Institutional care	10 (67%)	5 (33%)	0.003
Place of rehabilitation *			
Own home	18 (86%)	3 (14%)	
Rehabilitation facility	35 (95%)	2 (5%)	0.341
Rehabilitation setting *			
Individual	41 (89%)	5 (11%)	
Group	12 (100%)	0 (0%)	0.573

* Missing data from one patient.
